# Production and comparison of structural, thermal and physical characteristics of chitin nanoparticles obtained by different methods

**DOI:** 10.1038/s41598-024-65117-x

**Published:** 2024-06-25

**Authors:** Neda Moshtaghi Farokhi, Jafar Mohammadzadeh Milani, Zeinab Raftani Amiri

**Affiliations:** 1 Department of Food Science and Technology, Sari Agricultural Sciences and Natural Resources University, Sari, Mazandaran Iran; 2 Department of Food Science and Technology, Sari Agricultural Sciences and Natural Resources University, P.O. Box. 578, Sari, Mazandaran Iran

**Keywords:** Chitin, Nanocrystal, Nanofiber, Tempo, Grinding, Biochemistry, Nanoscience and technology, Techniques and instrumentation

## Abstract

This study examined the impact of acid hydrolysis, tempo oxidation, and mechanical grinding on the physical, thermal, and structural properties of α-chitin nanocrystals and nanofibers. The manufacturing methods could influence the diameter, functional groups, and crystal patterns of the resulting nanoparticles. Analysis of the DLS results revealed that the size of acidic nanocrystals were smaller and showed improved dispersibility. The XRD patterns indicated that the chemical and mechanical treatments did not alter the crystalline arrangement of the α-chitin. FT-IR spectra analysis revealed that the chemical and mechanical methods did not affect the functional groups of the nanoparticles. DSC results showed that the nanoparticles had good thermal stability up to 400 °C, and it was found that the nanofibers had better thermal resistance due to their longer length. In the FE-SEM images, the nanoparticles were observed as fiber mats with a length of more than 100 nm. It was also found that the diameter of the nanoparticles was less than 100 nm.

## Introduction

The focus on natural foods and drinks containing plant and animal compounds, as well as concerns about environmental problems caused by packaging made from petroleum derivatives, has led to a growing demand for natural materials in food and pharmaceutical systems^1^. Emulsions are one type of food system, consisting of a mixture of two or more immiscible liquids, and their stabilization is typically achieved by adding surfactants^2^. Pickering emulsions stabilized with natural micro- or nanoparticles (NPs) are superior and more stable than emulsions containing conventional surfactants^3^. Conversely, biodegradable food packaging films have weaker mechanical and barrier properties than polyethylene packaging. However, the addition of natural NPs to these films has been shown to have a positive effect on their mechanical, thermal, and barrier properties^4^.

Natural NPs are categorized into three groups: (1) proteins, such as soy protein, water-insoluble zein, and quinoa protein; (2) polysaccharides, such as modified starch, chitin, and cellulose; and (3) protein-polysaccharide mixtures, such as zein-chitosan and zein-acid tonic^5^. Chitin is one of the most abundant biopolymers in nature and the second most abundant biopolymer after cellulose^6^. Chitin is a natural polysaccharide found in the shells of crustaceans, such as crabs and shrimp, and the cell walls of fungi. In addition to its special properties such as high biodegradability, renewability, and non-toxicity^7^, chitin also has antibacterial and anti-allergic properties^8^. Chitin contains crystalline and amorphous components. Chitin NPs are produced in two forms: nanocrystals and nanofibers^9^. Chitin nanocrystals can be produced through acid hydrolysis, tempo oxidation, partial distillation, and ionic liquids. Meanwhile, chitin nanofibers can be produced through mechanical grinding, ultrasonic techniques, and electrospinning methods ^10,11^.

Several studies have been conducted on emulsions containing natural (NPs), showing that pickering emulsions prepared from chitin nanocrystals^12^ and chitin nanofibers^13^ exhibit good storage, physical, and thermal stability. Research on biodegradable starch films containing chitin NPs has demonstrated that nanocomposite films with chitin NPs have superior mechanical, barrier, and antifungal properties compared to films without NPs^14^. Research on gelatin films containing chitin nanofibers has shown that the presence of these particles increases the thermal and mechanical stability, as well as the water vapor resistance, compared to pure gelatin films^15^.

In this study, chitin NPs were produced using acid hydrolysis, tempo oxidation, and mechanical grinding methods. The study aimed to investigate and compare the impact of different nanoparticle production methods on their physical, chemical, thermal, and structural characteristics. This information will be valuable for researchers working in the field of biodegradable packaging films or natural emulsions.

## Results and discussion

### Carboxylate content and efficiency of tempo-oxidized chitin

**Figure 1:**
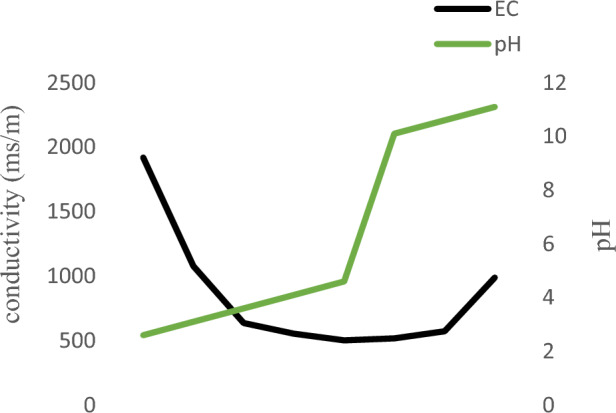
Conductometric titration and pH curves of TNC.

The carboxylate content of nanocrystals obtained from Tempo oxidation was determined using electrical conductivity (EC). When the titration started with a pH of 2.6, the EC decreased. This decrease continued until reaching a pH of 4.7. As the titration continued, the pH suddenly increased to 10. From this point onward, as the pH increased, the EC also increased (Fig. 1). The carboxylate content, as determined by electrical conductivity titration, was 1.1 mmol/g. After weighing chitin powder used in various methods of producing nanoparticles, and nanoparticle powder, it was determined that the Tempo method yielded more nanoparticles compared to other methods, with a yield of approximately 95%.

### Particle size and particle size distribution

The particle size, zeta potential, and dispersibility of NPs in an aqueous medium were measured by preparing a dispersion from the NPs powder. The size of the nanocrystals and nanofibers in the dispersion was then determined using DLS. The size, polydispersity index (PDI), and zeta potential results of the NPs are presented in Table 1. According to the results of DLS, The nanoparticles obtained through the acid hydrolysis method exhibited a smaller size compared to the nanoparticles produced through the tempo oxidation (in terms of diameter) and mechanical (in terms of length) methods. As a result, they were better dispersed in the dispersion and contributed to a more transparent appearance, as confirmed by visual observations. The PDI index is a parameter used to define the particle size distribution, expressed as a dimensionless number indicating molecules of approximately the same size. The PDI value may vary from 0.01 (mono-dispersed particles) to 1. However, values below 0.5 indicate a more uniform particle size distribution^16^. The PDI results indicated that the NPs produced by each of the methods had almost the same size. Zeta potential shows the relationship between the charged layers in the dispersion and the stability of the dispersion. The zeta potential above + 30 and below -30 shows the stability of the dispersion system^17^. The zeta potential results confirmed the good stability of the NPs dispersion.

**Table 1:** Particle size, PDI, and zeta potential of nanocrystals and nanofibers.

Nanoparticles	Particle size (nm)	PDI	Zeta potential (mv)
Acidic nanocrystal	${159.7}^{\text{b}}$	${0.317}^{\text{c}}$	${+33.4}^{\text{b}}$
Tempo nanocrystal	${187}^{\text{c}}$	${0.002}^{\text{a}}$	${-32.1}^{\text{a}}$
Nanofiber	${110}^{\text{a}}$	${0.024}^{\text{b}}$	${+43.1}^{\text{c}}$

Similar letters in each column indicate no significant difference between values (p ˂0.05).

### XRD

**Figure 2:**
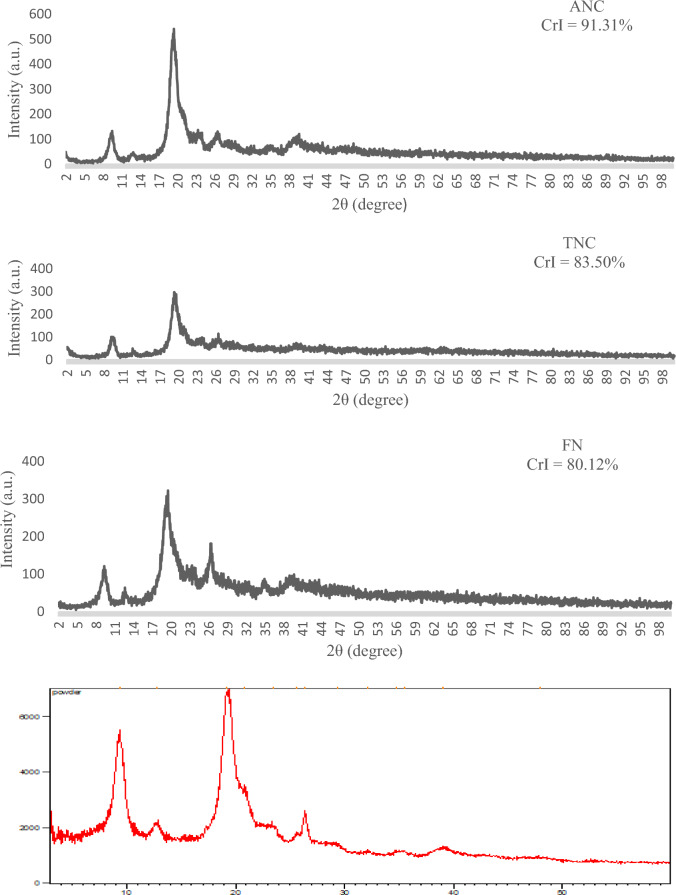
X-ray diffraction patterns of the pure chitin and produced chitin nanoparticles.

X-ray diffraction was used to determine the crystallinity index of the produced NPs. Figure 2 shows the diffraction patterns of pure chitin and NPs produced after physical and chemical treatments. Chitin indicated diffraction peaks at 2θ of 9.37°, 12.79°, 19.24°, 20.84°, 23.5°, and 26.35°, which corresponded to the 020, 021, 110, 120, 130, and 013 planes, respectively. The peaks for ANC treated were also detected (at 2θ = 9.48°, 12.88°, 19.44°, 20.01°, 23.32°, and 26.52°), as well as for TNC (at 2θ = 9.44°, 12.6°, 19.32°, 20.04°, 23.8°, and 26.44°), and NF (at 2θ = 9.32°, 12.64°, 19.52°, 20.01°, 23.36°, and 26.32°). These peaks are typical characteristics of α–chitin. The observed patterns showed that the chemical and mechanical treatments used to produce ANC, TNC, and NF did not alter the crystalline arrangement of α-chitin^18^. The increase in the intensity of the main crystalline diffraction peak at 19.4° was due to the increase in crystallinity after removing the amorphous parts. The crystallinity indexes (CrI) for chitin, ANC, TNC, and NF treated were 64.28%, 91.31%, 83.50%, and 80.12%, respectively. In general, the high crystallization index of the two NCs and NF respectively indicates the preservation of the crystal integrity of NF and NCs after mechanical and chemical processes.

### DSC

**Figure 3:**
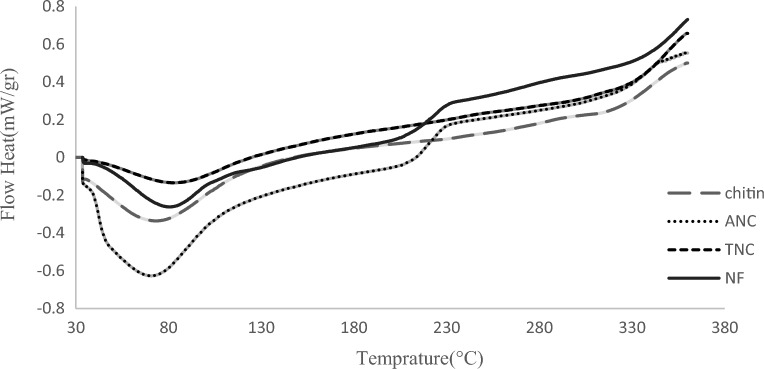
. DSC thermograms of the pure chitin, ANC, TNC and, NF.

Chitin has good thermal resistance due to its resistant mass structure, which is a result of the micellar structure caused by hydrogen bonds, including acetamide groups^19^. Chitin demonstrates excellent thermal stability due to its strong bulk structure, however, Weight loss and the main destruction of chitin structure and chitin NPs occur at temperatures below 400 °C^20^. The thermal stability of chitin and NPs was monitored up to 400 °C. Figure 3 depicts the DSC data of chitin, ANC, TNC, and NF. The endothermic peaks observed for chitin and NPs below 130 °C can be attributed to water evaporation. Degradation and thermal decomposition, as well as the release of volatile compounds, occurred at temperatures between 220 and 330 °C. Weight loss was more pronounced in ANCs due to their smaller size and higher surface-to-volume ratio. NFs are more thermally stable, which is related to the difference in their particle size (particle length). NFs are more thermally stable, which is related to the difference in their particle size (particle length) ^21^.

### FT-IR

**Figure 4:**
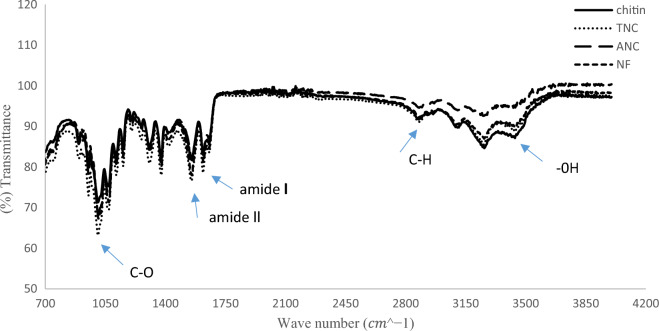
FTIR spectroscopy of chitin and chitin nanoparticles.

FT-IR analysis was used to study the functional group changes of chitin, chitin nanocrystals, and nanofibers (Fig. 4). The stretching band at 1030 ${\text{cm}}^{-1}$ was attributed to the C-O stretching vibration of the chitin skeleton and NPs. Absorption peaks at around 1560 ${\text{cm}}^{-1}$ and 1655 ${\text{cm}}^{-1}$, characteristic bands of chitin, were identified in all three nanoparticle spectra. These bands corresponded to amides II and I, respectively, and the sharper peaks in TNC indicated that the NaOH treatment causes partial acetylation^22^. The peak at around 1655 ${\text{cm}}^{-1}$ was related to the intramolecular hydrogen bond of acetylamide, which was associated with α-chitin (Pereira, Muniz, & Hsieh, 2014). C-H and -OH stretching bands were observed at around 2880 ${\text{cm}}^{-1}$ and 3480 ${\text{cm}}^{-1}$ respectively^23^. FT-IR data showed that chemical and mechanical treatments did not change the chemical nature and shape of α-chitin.

### FE-SEM

**Figure 5:**
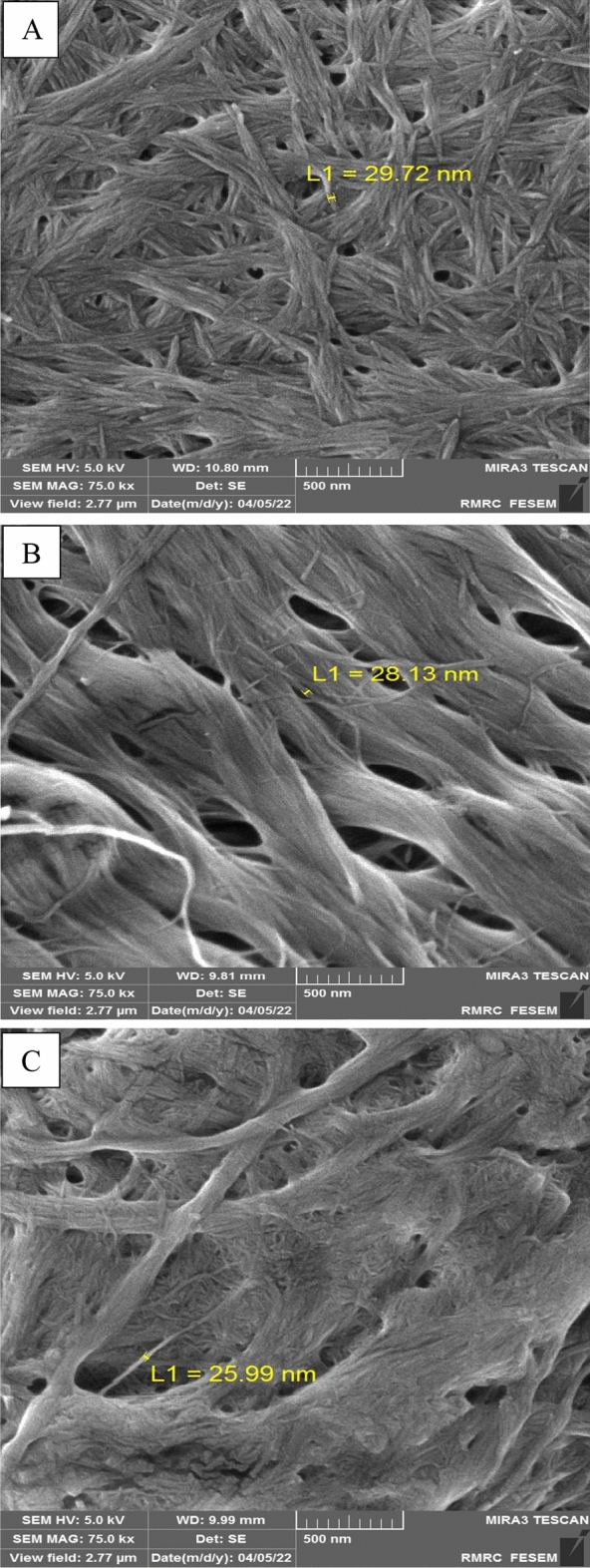
FE-SEM images: ANC (**A**), TNC (**B**), NF (**C**).

The FE-SEM was used to observe the microscopic structure of the produced NPs. The length of the nanocrystals and nanofibers was more than 100 nm, and their width was less than 100 nm. (Fig. 5). The microscopic images did not reveal a significant difference in the diameter of nanoparticles (p < 0.05), and all three NPs had a diameter below 30 nm. The FE-SEM images of ANC, TNC, and NF showed fiber mat shapes. It appears that the NPs stick together because of their high specific surface area, which results in the formation of strong hydrogen bonds between them, leading to the creation of long fibers through the process of freeze-drying^24^.

## Materials and methods

### Materials

Pure chitin flakes (deproteinized, decolorized, and demineralized) from shrimp skin were purchased from Nano Novin Polymer Co (Iran). All consumables, including hydrochloric acid, tempo, sodium bromide, sodium hypochlorite, ethanol, and sodium hydroxide, were prepared using the Merck brand.

### Production of chitin nanoparticles

#### Production of nanocrystals by acid hydrolysis method

Chitin was hydrolyzed with hydrochloric acid (3N) for 90 min at 90 °C. To produce nanocrystals, the hydrolysis process involved intense stirring with a stirrer set at 100 rpm^25^. The resulting suspension was then diluted with cold distilled water to prevent further hydrolysis. After that, the suspension was centrifuged twice at 4000 rpm for 15 min to remove excess acid. The resulting solution was dialyzed for five days using a dialysis bag (12–14 kDa) until a constant pH of 5–6 was achieved^26^. The suspension obtained was then ultrasonicated (300 W) for 20 min with two-second intervals. Finally, the samples were freeze-dried to obtain chitin acidic nanocrystal (ANC) powders^25,27^.

#### Production of nanocrystals by tempo oxidation method

The chitin powder was mixed with distilled water and stirred for 30 min to achieve a proper dispersion. After that, Tempo (0.1 mmol/10 g chitin) and sodium bromide (1 mmol/g chitin) were added to the dispersion. The oxidation of chitin by 2, 2, 6, 6-tetramethylpiperidine-1-oxyl (TEMPO) was initiated by adding a sodium hypochlorite solution (1–10 mmol/g chitin) ^14,22^. Dispersion titration was conducted at room temperature by adding NaOH (0.5 M) solution continuously until the pH reached 10. Once no more NaOH was being consumed and the pH remained stable at 10, the reaction was halted by adding a small amount of ethanol. The suspension was subsequently centrifuged at 10,000 rpm for 30 min and rinsed with ethanol. This procedure was repeated five times^23^. The dispersion was separated by washing and centrifugation, removing the soluble part containing neutralized carboxyl, while the insoluble part containing nanocrystals remained. The samples were then freeze-dried, yielding tempo nanocrystal (TNC) powder.

#### Production of chitin nanofibers

A mechanical mill was used to produce the nanofibers. First, a 1–3% chitin dispersion was prepared in water, and the pH was adjusted to approximately 3 by adding acetic acid. This was done to cationize the amine groups on the surface of the fibers to create an electrostatic repulsion force. The dispersion was mixed using a mixer for 10 min, and then a grinder (MKCA6.2, Masako Co, Japan) at 2500 rpm was circulated for three hours. The resulting dispersion was centrifuged to remove as much excess water as possible. The samples were freeze-dried to obtain chitin nanofiber (NF) powders^13^.

### Measurement and characterization

#### Determination of carboxylate content

The carboxylate content of tempo-oxidized chitins was determined using the electrical conductivity titration method. First, TNC powder (0.1 g) was mixed with water (100 mL) and a small amount of 0.1 M NaOH to adjust the pH to 9. The mixture was stirred for 30 min to prepare a well-dispersed slurry. Then, HCl (0.1 M) was added to the dispersion to set the pH in the range of 2.5–3.0. Titration was carried out at room temperature by adding NaOH (0.01 M) to the dispersion at a rate of 0.1 ml/min until reaching a pH of 11 using a pH–Stat titration system^23^. The resulting electrical conductivity and pH curves provided information about the carboxylate content.

#### Dynamic light scattering (DLS)

The particle sizes and zeta potential of the NPs' aqueous suspensions were measured using dynamic light scattering. The sizes of the nanocrystals and nanofibers were determined using DLS (Microtrac MBR, USA) at room temperature. Deionized water was used as the dispersant. The refractive indices of water and chitin were 1.33 and 1.61, respectively^5^.

#### X-ray diffraction (XRD)

The samples were analyzed using X-ray diffraction (XRD) with an X-ray diffractometer (XPERT-PRO) and Cu-Kα radiation (λ = 0.1541 nm, voltage = 40 kV, and filament current = 40 mA). The XRD patterns of the chitin and NPs samples were recorded at a scan rate of 1°/min in the 2θ angle range of 5°–60° at room temperature. The crystallinity index was calculated using the Eq. (1):

1 $$CrI  \, \%= {\text{I}}_{110} -  {\text{I}}_{\text{am}} /{\text{I}}_{110}\times 100$$

where ${\text{I}}_{110}$ shows the maximum intensity of diffraction at 2θ = 19$^\circ$ and ${\text{I}}_{\text{am}}$ indicates the intensity of amorphous parts (2θ = 12$^\circ$ degrees) ^28,29^.

#### Differential scanning calorimetry (DSC)

The thermal properties of chitin, nanocrystals, and nanofibers were measured using a differential scanning calorimeter (Sanaf 500, Iran). DSC is a widely used method for studying the thermal behavior of materials. For the testing, 15 mg of chitin powder and NPs were scanned from 30 to 400 °C at a rate of 10 °C/min under a flow of liquid nitrogen at 20 ml/min^12^.

#### Fourier transform infrared spectroscopy (FT-IR)

FT-IR spectra were obtained using a Fourier transform infrared spectrophotometer (Cray 360, USA) to analyze the alterations in the functional groups of the materials resulting from various chemical and physical treatments. The FT-IR spectra of chitin and chitin NPs were captured within the wavelength range of 400–4000 ${\text{cm}}^{-1}$ with a resolution of 4 ${\text{cm}}^{-1}.\text{ The}$ conditioned chitin, chitin nanocrystal, and nanofiber samples were transformed into disks using KBr salt at a ratio of 1:100 and then inserted into the instrument^30^.

#### Field emission-scanning electron microscopy (FE-SEM)

A field-emission scanning electron microscope was utilized to examine the morphology of chitin, nanocrystals, and chitin nanofibers. The chitin and NPs were coated with gold to prevent charging and then transferred to the device. The images were captured at a magnification of 75.0 KX. The FE-SEM (TESCAN (MIRA 3 LMU)) was operated at a voltage of 5.0 kV^31^.

### Statistical analysis

Statistical analysis was performed using SPSS (SPSS Inc., Chicago, IL, USA). Duncan’s test was used to determine significant differences (p < 0.05) between means.

## Conclusions

The study of dispersions prepared from chitin NPs revealed that the NPs obtained through acid hydrolysis had a smaller size, resulting in a larger contact surface in the ANC. This improved their dispersibility and increased the transparency of the dispersion. The examination of NPs showed that the production of nanocrystals using the tempo method had a higher efficiency (above 90%) than those produced using the acid method. Additionally, deacetylation occurred less in TNC compared to ANC. After examining the X-ray diffraction pattern, it was determined that the crystal pattern of alpha-chitin remained unchanged when using chemical and mechanical methods to produce NPs. Additionally, it was observed that the ANC had the highest crystallinity index, and the increase in intensity of the main crystalline diffraction peak at 19.4° was attributed to the enhanced crystallinity resulting from the removal of amorphous components. The FT-IR results revealed that the use of chemicals and mechanical grinding to produce nanoparticles did not alter the functional groups and structure of alpha-chitin. DSC curves confirmed the strong thermal resistance of the NPs, attributed to the robust intramolecular and intermolecular bonds in chitin and NPs. Furthermore, NFs demonstrated superior thermal stability compared to other NPs, mainly due to their larger size. Electron microscope images revealed a fibrous appearance in the NPs, likely resulting from hydrogen bonding between molecules during the freeze-drying process. These images showed that the NFs had a smaller diameter than the other two NPs, despite being longer in length. Based on the results of experiments conducted on nanocrystals and nanofibers, it was found that the methods used to produce nanoparticles did not have a significant impact on their physical, structural, and thermal characteristics.

## Data Availability

The datasets used and/or analysed during the current study available from the corresponding author on reasonable request.
